# Novel Viruses Found in *Antricola* Ticks Collected in Bat Caves in the Western Amazonia of Brazil

**DOI:** 10.3390/v12010048

**Published:** 2019-12-31

**Authors:** Anne-Lie Blomström, Hermes R. Luz, Pontus Öhlund, Matthew Lukenge, Paulo Eduardo Brandão, Marcelo B. Labruna, Mikael Berg

**Affiliations:** 1 Department of Biomedical Sciences and Veterinary Public Health, Swedish University of Agricultural Sciences, Box 7028, 75007 Uppsala, Sweden; pontus.ohlund@slu.se (P.Ö.); lukengemat@gmail.com (M.L.); mikael.berg@slu.se (M.B.); 2 Departments of Preventive Veterinary Medicine and Animal Health, School of Veterinary Medicine and Animal Science, University of São Paulo, São Paulo P.O. 05508-270, Brazil; hermesluz@usp.br (H.R.L.); paulo7926@usp.br (P.E.B.); labruna@usp.br (M.B.L.); 3 Vector Biology Unit, Division of Entomology, Uganda Virus Research Institute, Entebbe P.O. Box 49, Uganda

**Keywords:** Ticks, *Antricola delacruzi*, virus, metagenomics, high-throughput sequencing, *Nairoviridae*, bats

## Abstract

In this study, we describe the viral composition of adult *Antricola delacruzi* ticks collected in a hot bat cave in the state of Rondônia, Western Amazonia, Brazil. *A. delacruzi* ticks, are special, compared to many other ticks, in that they feed on both bats (larval blood feeding) and bat guano (nymphal and adult feeding) instead of feeding exclusively on vertebrate hosts (blood feeding). Considering this unique life-cycle it is potentially possible that these ticks can pick up/be infected by viruses not only present in the blood of viremic bats but also by virus shed through the bat guano. The viral metagenomic investigation of adult ticks showed that single-stranded negative-sense RNA viruses were the dominant group of viruses identified in the investigated ticks. Out of these, members of the *Nairoviridae* family were in clear majority constituting 88% of all viral reads in the data set. Genetic and phylogenetic analyses indicate the presence of several different orthonairoviruses in the investigated ticks with only distant relationship to previously described ones. In addition, identification of viral sequences belonging to *Orthomyxoviridae*, *Iflaviridae*, *Dicistroviridae*, *Polycipiviridae*, *Reoviridae* and different unclassified RNA viruses showed the presence of viruses with low sequence similarity to previously described viruses.

## 1. Introduction

Vector-borne diseases are of major concern for many human and animal populations and there are many viruses of great importance that are maintained and spread by blood-sucking arthropods, such as mosquitos and ticks [1]. Many of these arboviruses are maintained in nature by a natural vertebrate reservoir, such as birds, rodents and wild boars. One other potential reservoir for arboviruses, that is known to harbour many known and potentially emerging viruses, are bats. Most viral families can be found in bats, but particular viral richness lies in the families of *Flavi*-, *Bunya*- and *Rhabdoviridae* [2]. Bats seem to be able to tolerate potential zoonotic viruses in greater numbers [3] and can therefore act as a reservoir for many future emerging diseases. The question regarding if bats can act as reservoirs for arboviruses is being discussed [4] and so far no consensus has been reached but it is at least known that bats and various blood-sucking arthropods share ecological niches and that there are many arthropods that feed on bats.

After mosquitoes, the second most important arthropod vector for arboviruses of public health concern is ticks and they have been shown to harbour viruses belonging to different families, such as *Rhabdoviridae*, *Reoviridae*, *Flaviviridae* and to various orders, such as, *Bunyavirales* and *Mononegavirales* [5,6,7]. There are up to one thousand tick species known and these have been classified into three families, Argasidae, Ixodidae and Nuttalliellidae [8]. Examples of tick-borne viruses of importance for animal and/or human health are African swine fever virus transmitted by *Ornithodoros* spp. [9], Crimean-Congo hemorrhagic fever virus (CCHFV) transmitted by *Hyalomma* ticks [10] and tick-borne encephalitis virus transmitted by *Ixodides* ticks [11]. As mentioned earlier, the ecology of these viruses, normally includes the circulation of the virus between a reservoir animal and the tick species. For example, in the case of CCHFV the main viral reservoir is the tick itself with several amplifying hosts, such as hares, deer and domestic livestock. Humans can be infected via tick bites or when handling infected animals [10].

In the neotropical region, bats are known to sustain a variety of ticks of at least three genera of the Argasidae family (soft ticks): *Antricola*, *Nothoaspis, Ornithodoros* [12,13]. Of particular interest is the genus *Antricola,* composed of 17 species, all inhabiting hot caves supporting thousands of insectivorous Mormoopidae bats, especially of the genus *Pteronotus* [13,14]. As opposed to the majority of tick species, which are blood-feeding through all post-embryonic stages (larvae, nymphs, adults), only the larval stage of *Antricola* is hematophagous. The subsequent stages of nymphs and adults are non-parasitic, and are considered to be “guanophagous”; i.e., they are supposed to feed on bat guano, which is very abundant in these hot caves [15]. Interestingly, bat guano is a food source consisting of an iron and chitin-rich substrat that harbours a rich microbiota that grows on it [16,17].

Recently, the viral diversity of *Rhipicephalus microplus* in southern Brazil was determined [5]. Apart from that study, very little is known about the virome of ticks in other parts of Brazil and in other tick species. Therefore, in this paper, we investigated the virome of adult *Antricola* ticks collected in a hot bat cave in the state of Rondônia, Brazilian Amazonia, and through this study, we identified a large number of novel viral sequences from various families.

## 2. Materials and Methods

### 2.1. Collection of Ticks

In July 2017, we collected 180 adult *Antricola delacruzi* ticks from bat guano in a hot cave in Porto Velho Municipality, state of Rondônia, western Amazon, northern Brazil. The cave structure was composed by a hot and humid ecosystem that generates high temperature (33–38 °C) and an atmosphere rich in nitrogen compounds throughout the year. The guano inside the cave was abundant (approximately 10–30 cm in depth), moist, and sticky, as previously described [15,18,19]. Collected ticks were brought live to the laboratory.

### 2.2. Nucleic Acid Extraction

The 180 ticks were washed twice with sodium hypochlorite before being washed in sterile water. Thereafter, the ticks were homogenised in pools of five in TRIzol (Thermo Fisher, Waltham, MA, USA) using Precelly ck14 tubes (Bertin Technologies, Montigny-le-Bretonneux, France) prior to RNA extraction using a combined protocol for TRIzol and Genjet RNA extraction kit (Thermo Fisher, Waltham, MA, USA) as described previously [6]. The RNA was eluted in 40 μL EB and DNA was removed using the RNase-Free DNase Set (QIAGEN, Hilden, Germany). In addition, ribosomal RNA was depleted through the use of Ribo-Zero Gold (epidemiology) rRNA Removal Kit (Illumina, San Diego, CA, USA) according to the manufacturer’s instructions.

### 2.3. Nucleic Acid Amplification and Sequencing

The remaining RNA was used to obtain cDNA and amplified through a Single-primer isothermal amplification (SPIA) approach using the Ovation RNA-seq v2 kit (NuGEN, San Carlos, CA, USA) according to the manufacturer’s instructions. The SPIA products were purified using Genjet PCR purification kit (Thermo Fisher, Waltham, MA, USA) according to the manufacturer’s instructions before being sequenced at SciLifeLab/Genome Center (Uppsala, Sweden) using the Ion S5 XL system (Thermo Fisher, Waltham, MA, USA) and a 530 chip.

### 2.4. High-Throughput Sequencing (HTS) Data Analysis

The raw HTS data was imported to the CLC genomic workbench (version 11) and trimmed based on quality (Q = 20) and length (≥50). The trimmed reads were annotated through blastx (*E*-value ≤ 0.0001) using Diamond (version 0.9.10) [20]. The diamond results were visualized using Megan (ver. 6.13) [21]. The dataset containing the raw data have been deposited in GenBank under the SRA accession: PRJNA577110.

### 2.5. Viral Genetic Analyses

In order to be able to analyse longer viral sequences, de novo assembly using CLC genomic workbench (version 11) was performed to create longer contigs. This was done both on reads belonging to, for example, a specific family extracted from MEGAN as well as on the complete trimmed read data set. For the viral contigs studied in more detail, open reading frames were predicted using ORFfinder (https://www.ncbi.nlm.nih.gov/orffinder/) and blastp was used to identify related viruses and determine their similarity to each investigated contig. Muscle alignments and the phylogenetic analyses were performed using MEGA7 [22], using the Maximum Likelihood method based on the JTT matrix-based model [23]. The percentage of trees in which the associated taxa clustered together is shown next to the branches of each tree. Initial tree(s) for the heuristic search were obtained automatically by applying Neighbour-Join and BioNJ algorithms to a matrix of pairwise distances estimated using a JTT model, and then selecting the topology with superior log likelihood value. The trees are drawn to scale, with branch lengths measured in the number of substitutions per site. The analysed sequences were deposited in GenBank with the accession numbers MN560621–MN560637.

## 3. Results

### 3.1. Sequencing Output

**Figure 1:**
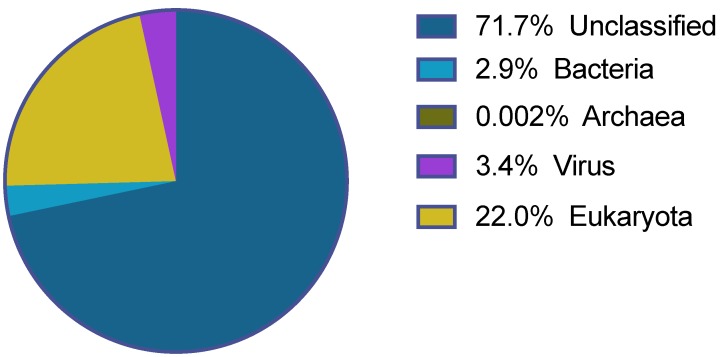
Classification of the reads obtained from the Ion S5 XL sequencing.

In total, 27,195,787 sequencing reads were obtained after the Ion S5 XL sequencing of the 180 *Antricola delacruzi* ticks. The majority of these were of good quality and after size and quality trimming 25,575,445 reads with an average length of 302 nucleotides (nt) remained. The majority of the reads (>70%) remained unclassified after the blastx analysis. Of the classified reads most were eukaryotic, while the remaining mapped to bacteria, archaea and virus (Figure 1).

### 3.2. Viral Community

The blastx analysis of the 25,575,445 trimmed reads showed that 3.4% of all the sequencing reads (i.e., 854,361 reads) were of viral origin. The reads classified to 47 viral families, however, for most of these families few reads were found. It was only eight viral families that had more than 500 reads assigned to them, shown in Table 1. In addition, a number of viral reads could not be assigned to a family and was marked as unclassified.

### 3.3. Single-Stranded Negative-Sense RNA Viruses

As shown in Table 1, single-stranded negative-sense RNA viruses were the dominant viral group in the investigated ticks. Out of these members of the *Bunyavirales* were in clear majority constituting 89.4% of all viral reads in the data set but also the families *Rhabdoviridae* and *Orthomyxoviridae* were found. Most Bunyavirales reads classified as being members of the *Nairoviridae* family, but also potential *Peribunyaviridae* members were found. However, investigating the reads that, through Diamond and Megan analysis, were classified as *Peribunyaviridae* members revealed that most of these showed closest similarity to orthonairoviruses and, thus, the focus in the section below will be only on the *Nairoviridae* family. In addition, a low number of reads (n. 7) were categorised as unclassified *Bunyavirales*. Considering the abundance of Bunyavirales reads de novo assembly was performed and the analysis focused on contigs (n. 1065) only.

#### 3.3.1. Nairoviridae

Reads belonging to members of the *Nairoviridae* family (tri-segmented viruses) were in clear majority, constituting 88.2% of all viral reads in the data set. From the 1065 *Bunyavirales* contigs, 1055 classified as *Nairoviridae*. All the segments (S, M and L) were represented although at different proportions. The L-segment, encoding the RNA-dependent RNA polymerase (RdRp), was in majority (55.6% of the contigs), while 34.5% and 9% of the contigs represented the M- and S-segments, respectively. The contigs matching to an individual segment together spanned across most of the entire protein coding sequence, however, they did not fully assemble despite some of the contigs overlapping suggesting that they may belong to several different orthonairoviruses.

**Figure 2:**
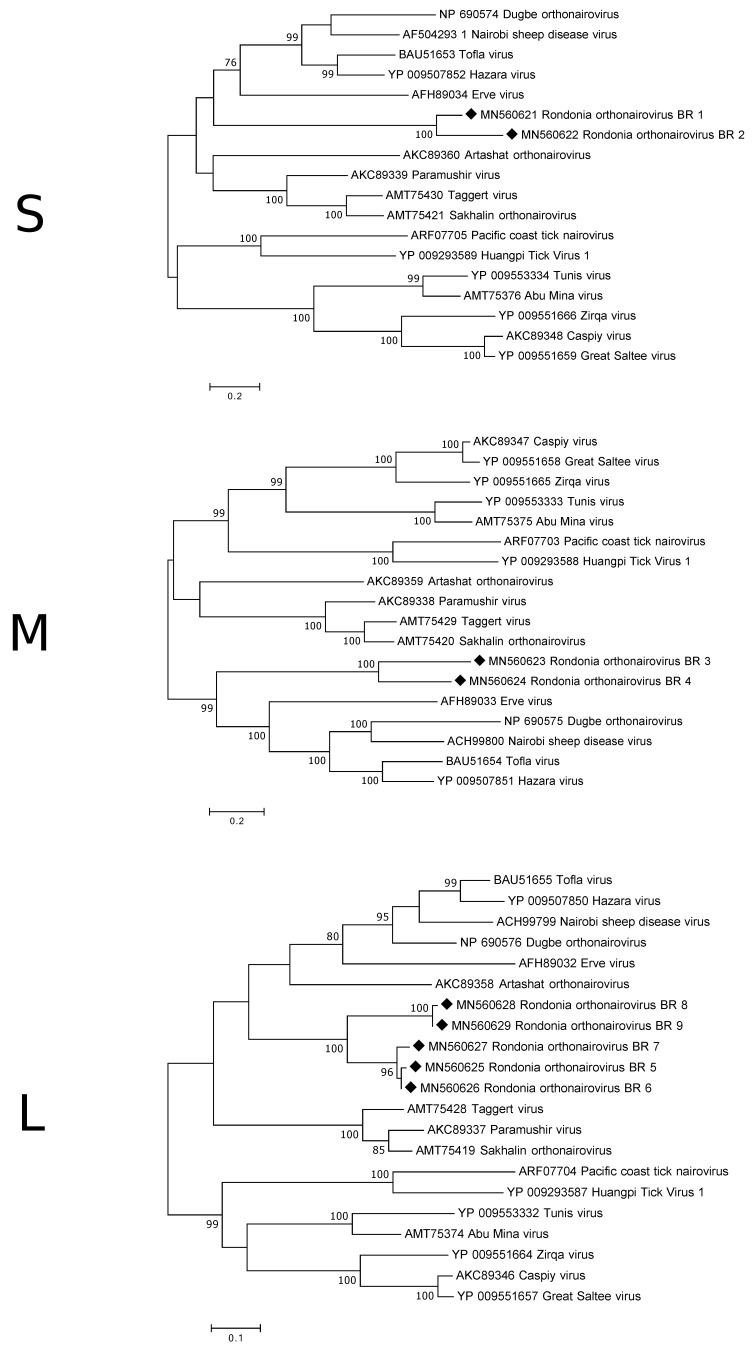
Phylogenetic analysis of the S-, M- and L-segment of orthonairoviruses. The map was inferred using the Maximum Likelihood method with a bootstrap of 500. There was a total of 238 (S-segment), 1101 (M-segment) and 208 (L-segment) positions, respectively, in the final data set. The black diamond marks the sequences obtained from this study.

Five of the S-segment contigs were long enough to cover at least approximately half of the segment and were, thus, analysed more in detail. The longest contig was 2005 nt in length and is believed to contain the complete coding region of a 500 a.a. (amino acid) long nucleocapsid protein (NP). The other four contigs only covered approximate 50% of the coding region, with two of them covering the first half and the other two the second half. Three of the contigs show >99% a.a. similarity to each other and only 70% similarity to the other two suggesting that there are at least two different orthonairoviruses in the data set. Although, there are different orthonairoviruses in the investigated samples, based on the S-segment, they are still genetically closer to each other than to those available in GenBank as the a.a. similarity to the NP of previously described orthonairoviruses, such as CCHFV, Dugbe orthonairovirus and Erve virus, ranged between 35–44%. This was also confirmed by the phylogenetic analysis (Figure 2). 

Two of the contigs matching the M-segment appear to have the complete/near complete coding sequence for the glycoprotein precursor and were, therefore, analysed further. The two predicted protein sequences (1357 a.a. and 1263 a.a) showed, in the overlapping part, an amino acid similarity of 60%. The similarity to other orthonairoviruses, such as, *Artashat orthonairovirus*, Erve virus and *Thiafora orthonairovirus*, were 30–37%. Phylogenetically, the two glycoprotein sequences from this study grouped together on a clade closest associate with viruses such as Nairobi sheep disease virus, Hazara virus and Erve virus (Figure 2).

Unfortunately, no contig covered the entire L-segment. The expected size of the L-segment of orthonairoviruses is 7–12 kb, however, the longest contig was only 2720 nt. The protein sequences of the longer contigs were aligned and showed that in many parts of the RdRp they were nearly identical to each other, however, as for the other two segments there were differences observed. Phylogenetic analysis displayed a similar grouping as for the M-segment (Figure 2). The a.a. sequences obtained from the antricola ticks were 74–100% similar, while the similarity to the orthonairoviruses were 43–64%.

#### 3.3.2. Rhabdoviridae

**Figure 3:**
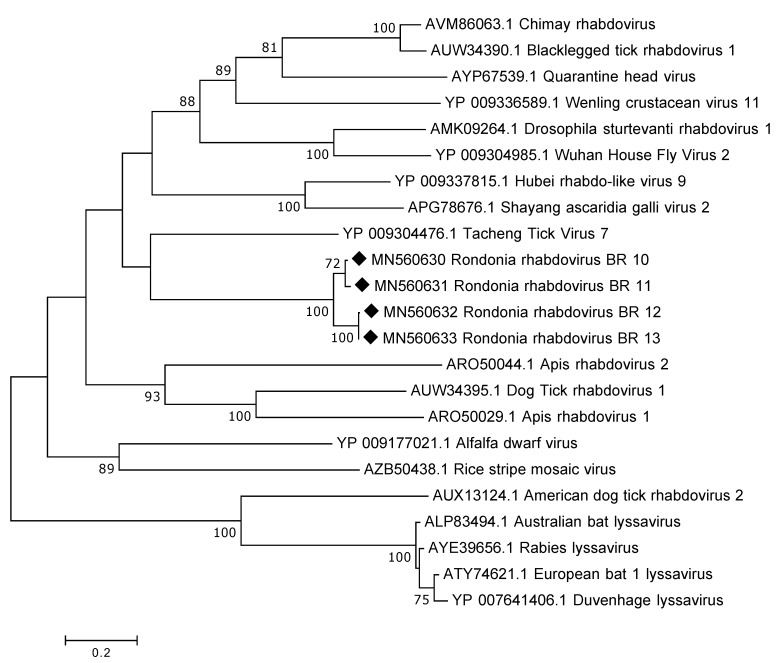
Phylogenetic analysis of the RdRp protein of rhabdoviruses. The map was inferred using the Maximum Likelihood method with a bootstrap of 500. There was a total of 492 positions in the final data set. The black diamond marks the sequences obtained from this study.

The genome of rhabdoviruses range between 11 and 15 kb in size, encoding five different proteins. No complete genome was recovered in this study; the longest contig obtained was 6787 nt in length. This sequence contained a larger portion of the L-gene and the predicted protein (RdRp) showed a distant relation to various rhabdoviruses, such as, Tacheng Tick Virus 7, Quarantine head virus and Chimay rhabdovirus, with a.a. identity of around 30%. Despite the distant relationship InterProScan could identify the following domains: Mononegavirales RNA-directed RNA polymerase catalytic domain and Mononegavirales mRNA-capping domain V. In total, there were seven contigs >1000 nt in length and all of them mapped partially to the L gene. In addition, one of the contigs also contained a region (1551 nt) prior to the L gene that we believe is coding for the glycoprotein. Sequence alignment of the overlapping RdRp protein sequences from the tick samples showed a similarity of 80–100%. As expected, the partial RdRp sequences from the Antricola ticks grouped phylogenetically together and it was also observed that they grouped away from lyssaviruses (Figure 3).

#### 3.3.3. Orthomyxoviridae

**Figure 4:**
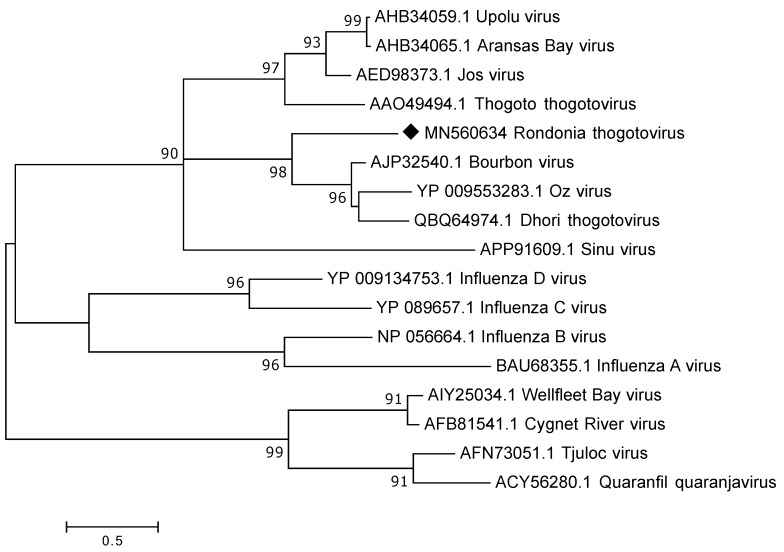
Phylogenetic analysis of the complete matrix protein of members of the *Orthomyxoviridae* family. The map was inferred using the Maximum Likelihood method with a bootstrap of 500. The black diamond marks the sequence obtained from this study.

All *Orthomyxoviridae* reads classified as thogotoviruses, a group of segmented viruses that are known to infect arthropods, such as, ticks as well as in some cases vertebrates. To analyse these reads further de novo assembly was performed and from the 17,553 *Orthomyxoviridae* reads only 40 reads remained un-matched while the remaining reads were assembled into 29 contigs ranging from 206 to 2369 nt in length. Unfortunately, the complete segments were not recovered through the data analysis but contigs and reads could be found that matched to all the proteins of the six expected segments i.e., to polymerase PB2, polymerase PB1, polymerase PA, glycoprotein (GP), nucleoprotein (NP) and matrix (M). However, for M the complete/near complete coding sequence was recovered, as were parts of the 3′UTR. The obtained M protein (246 a.a.) was divergent showing a protein sequence identity of around 45% to known thogotoviruses available in GenBank, such as, Bourbon-, Oz- and Dhori virus. Comparisons to other thogotoviruses indicate that potentially about 15–30 a.a. are missing at the 5′UTR. In the phylogenetic analysis our sequence grouped on a separate subclade of thogotoviruses (Figure 4). 

Despite the divergence of the M sequence, partial sequence conservation was observed in the UTR. The sequence AGCAATCCCAAGGGTTGCCTCT found in the 3′terminus (genomic orientation) is similar to that of other thogotoviruses, for example that of Dhori virus (AGCAATAACAAGCAGTACTAGA). Investigation of the other thogotovirus-related contigs showed that the protein coverage and a.a. identity varied between the proteins, as shown in Table 2.

### 3.4. Single-Stranded Positive-Sense RNA Viruses

Although single-stranded negative-sense RNA viruses were found in abundance also positive-sense RNA viruses were found in the ticks. Below follows a description of the viruses detected in the three main viral families found in this group, i.e., in *Iflaviridae*, *Dicistroviridae* and *Polycipiviridae*.

#### 3.4.1. Iflaviridae

**Figure 5:**
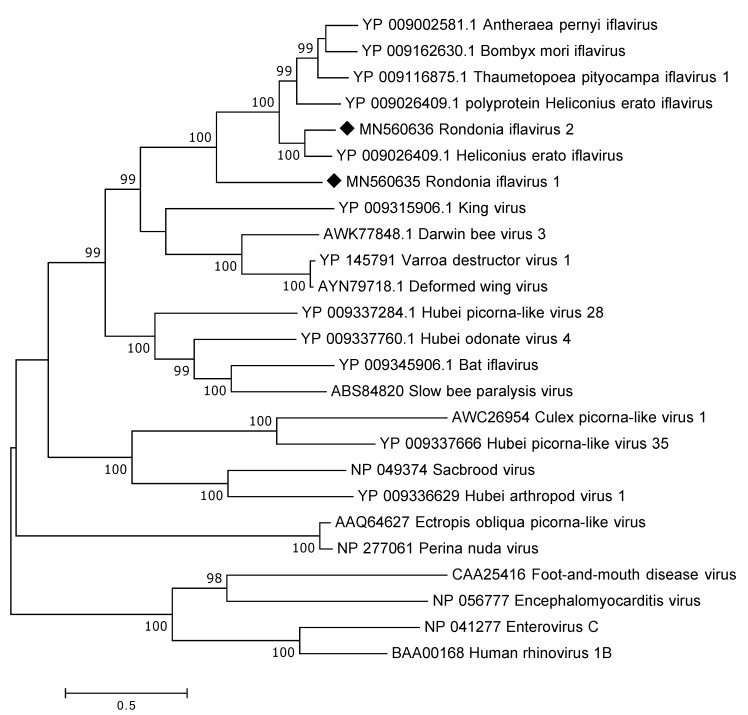
Phylogentic analysis of the polyprotein of iflaviruses, four members of the *Picornaviridae* are also included. The map was inferred using the Maximum Likelihood method with a bootstrap of 500. The black diamond marks the sequences obtained from this study.

By assembling all the *Iflaviridae* reads the complete polyprotein was recovered including parts of the UTR. The polyprotein is encoded by 8922 nt and, thus, consist of 2973 a.a. Protein blast analysis showed that the identified *Iflaviridae* polyprotein was highly divergent to presently known iflaviruses showing around 30–40% a.a. identity across the complete polyprotein. In addition, a near complete polyprotein of a second iflavirus was recovered from the data. The polyprotein is 2882 a.a. in length and is missing the 3′end. This iflavirus showed only a 39% a.a. sequence identity to the first one but was instead more similar to those iflaviruses available at GenBank showing a 73% a.a. sequence identity to Helicoverpa armigera iflavirus (YP_009344960). In the phylogenetic analysis (Figure 5), the identified viruses were grouped with iflaviruses found in different insects.

Further sequence analysis of the polyproteins confirmed the presence of several conserved regions including a domain that occurs in the capsid protein of picornaviruses and caliciviruses. In addition, three helicase domains, previously described by Koonin and Dolja (1993) [24], were identified between position 1640–1761 (iflavirus 1) and 1570–1689 (iflavirus 2). Similar to other iflaviruses and picorna-like viruses a protease domain was identified with the conserved motifs GxCG in position 2469–2473 (iflavirus 1) and 2422–2426 (iflavirus 2) and GxHxxG in position 2404–2409 (iflavirus 1) and 2439–2444 (iflavirus 2). In addition, eight conserved RNA-dependent RNA polymerase domains were identified in position 2640–2902 for iflavirus 1. As the complete polyprotein for iflavirus 2 was not recovered only domain I–VI was present in position 2676–2869.

#### 3.4.2. Dicistroviridae

The 10,224 reads mapping to the *Dicistroviridae* family classified to three genera: *Aparavirus* (n. 382), *Cripavirus* (n. 2866), *Triatovirus* (n. 6437). In addition, a portion of the reads were marked as being unclassified *Dicistroviridae* (n. 539). The genomes of members of the *Dicistroviridae* are approximately 8–10 kb in length, however, assembling the different genera reads only yielded shorter contigs. 

The longest *Aparavirus* contig was 1010 nt in length and matched with a high nucleotide identity (78–95%) to the capsid gene of various Acute bee paralysis viruses. A contig of 786 nt matched to another region of the capsid protein with similar identities. Also, contigs matching three regions of the replicase gene of the same virus were found. For these the nucleotide identity was around 80%. However, more divergent *Aparavirus* contigs were also found showing only 30–40% amino acid identity to characterised *Aparavirus* available in GenBank indicating that several *Aparavirus* may be present in the investigated ticks. The longest *Cripavirus* contig yielded a 757 a.a. long protein sequence showing highest protein identity (45–49%) to the non-structural protein of viruses such as, for example, Drosophila C virus and Cricket paralysis virus. Contigs matching the structural/capsid protein of the mentioned viruses to a similar protein identity were also identified. Also, in the *Triatovirus* contig set both structural and non-structural sequences were identified. The sequences matched to viruses such as, for example, Homalodisca coagulata virus 1, Triatoma virus and Himetobi P virus with the a.a. sequence identity varying from approximate 45–68%.

#### 3.4.3. Polycipiviridae

Only 1271 reads classified within this family, majority of these matched to viruses within the genus *Sopolycivirus*, a few to genus *Chipolycivirus* and a few were marked as unclassified *Polycipviridae*. The *Sopolycivirus* reads showed similarity to a number of viruses found in various insects, such as, for example, Lasius neglectus virus 1, Myrmica scabrinodis virus 1 and Solenopsis virus. Only shorter contigs were obtained and the protein sequence identity varied between 25–90%. All of the Chipolycivirus reads classified as Hubei chipolycivirus, through the blastx analysis, and more specifically to Hubei picorna-like virus 81 and 82. These are both picorna-like viruses that were identified and partially genetically characterised from insects through a large metagenomic study in China [25]. The protein sequence identity was, however, for both of these viruses low ranging between 22–66%. The unclassified reads showed closest similarity to two viruses, Linepithema humile polycipivirus 1 and Linepithema humile polycipivirus 2, identified in Argentine ants. The protein sequence identity was, as seen for the other *Polycipiviridae* viruses, most often low ranging from 23–60%.

### 3.5. Double-Stranded RNA Viruses

Although *Partitiviridae*, *Totiviridae* and unclassified double-stranded RNA viruses were identified through the blastx analysis the vast majority of double-stranded RNA reads (94.2%) belonged to *Reoviridae* and was, thus, analysed in more details. These viruses have 10–12 segments and have been found in a wide range of different hosts.

#### Reoviridae

Majority of the *Reoviridae* reads classified as rotaviruses and, thus, to further analyse the rotavirus reads identified in the data set the reads were de novo assembled and each contig were inspected to identify its protein sequence identity to rotaviruses present in GenBank. In addition, as rotaviruses have 11 segments coding for different proteins it was investigated if all the segments/proteins could be identified. The results are shown in Table 3 and as the contigs consistently mapped against human and porcine rotaviruses and one of the top hits were the adult diarrheal rotavirus strain J19 this strain was used in the comparison. In summary, all but one protein (NSP6) could be detected. Unfortunately, no complete coding sequence was recovered, and the coverage of the different proteins varied between 27.7–92.6%. The protein sequence identity varied between the different segments and the different positions in the protein and ranged between 49–94%. The fact that some of the contigs matched to the same position in certain proteins but had assembled to different contigs indicate that the sample contain at least two rotaviruses. 

In addition, two of the contigs obtained from the rotavirus de novo assembly did not map to different vertebrate rotaviruses, but instead showed closest similarity (39% and 43% on protein level) to the RNA-dependent RNA-polymerase of Fako virus (isolated from mosquitoes) and Rice gall dwarf virus, respectively. Also, among the reads that remained unmapped after the de novo assembly were those that showed similarity to different plant and insect *Reoviridae* viruses further indicating the presence of other rotaviruses from this family.

### 3.6. Unclassified Viruses

The 32,371 unclassified viral reads matched, with a varied protein sequence identity (approximate 25–70%), to a vast number of different viruses having mainly an arthropod or plant host. For many of these only a low number of reads matched each respective virus and will therefore not be discussed more in detail. However, for some viruses in the unclassified group a large number of reads were identified and, in some cases, long contigs could be retrieved when de novo assembled.

A 11,482 nt long contig yielded a 3770 long a.a. sequence. No stop codon was observed indicating that the contig is missing coding sequence the 3′ end. Through blastp analysis it was shown that the protein had closest similarity to various unclassified viruses such as Hubei picorna-like virus 55, Changjiang crawfish virus 6 and Rosy apple aphid virus. However, it should be noted that only 910 of the 3770 a.a. matched these viruses and then to a very low identity of around 23%. The part matching was in the predicted RNA-dependent RNA polymerase region. Together this indicates that this is a completely novel RNA virus. In addition, other longer contigs included a 6585 nt long sequence coding for a partial RdRp with closest a.a. similarities (30–37%) to viruses such as Ingleside virus and Hubei virga-like virus 14 and a 6342 nt sequence with a 1881 nt long ORF (no stop codon) coding for a protein showing 42% similarity to the first half of a hypothetical protein of Hubei coleoptera virus 2.

## 4. Discussion

It is estimated, by the World Health Organization, that 17% of all infectious disease are caused by vector-borne pathogens. Ticks have been somewhat overlooked as important viral arthropod vectors, as few tick-borne viruses of human and animal concern have been known. However, in the last decade several pathogenic tick-borne viruses have been detected showing their importance [26]. In this study, we identified numerous viruses present in adult *A. delacruzi* ticks collected from a hot bat cave in the Amazonia. These tick are different from most other tick species in that it is only the larvae that is hematophagous, i.e., feeding blood from the bats in the cave, while the other two stages (adults and nymphs) are “guanophagous” i.e., feeding on the bat guano on the ground of the cave [15,19]. Considering this unique cycle, it is possible that these ticks can pick up/be infected by viruses not only present in the blood of viremic bats but also by virus shed through the bat guano. It should be noted that as the whole body of the ticks were used it is not possible to determine if the viruses identified in this study are tick-borne or bat viruses. Also, as the bats in the cave are insectivorous the identified viruses could also be, for example, insect-viruses shed from the bats. 

The results show that a vast number of viruses, from different families and orders, are present in this special ecosystem of ticks and bats. One viral family did, however, stand out and that was the *Nairoviridae* belonging to the order *Bunyavirales*. Approximately, 88% of all viral reads in our data set were categorised as belonging to this family and among these sequences matching to all three segments (S, M and L) were identified. Most viruses in this family are tick-borne and are transmitted to different mammals (including bats), birds, fish and reptiles through the blood meal [27]. In addition, several viruses of veterinary and public health concern belong to this group and are known to cause mild to severe disease and sometime even death. Exampled of these known pathogenic orthonairoviruses transmitted by ticks are CCHFV, severe fever with thrombocytopenia syndrome virus, louping ill virus, Erve virus and Nairobi sheep disease virus. The nairoviral sequences identified through our study are clearly genetically different from those previously characterised as they only show 30–50% a.a. identity to other orthonairoviruses available in GenBank. They did, however, phylogenetically group together with viruses known to infect and cause disease in mammals and could, thus, potentially have similar properties. The results also indicate the presence of more than one orthonairovirus in the samples as there are variation between some of the overlapping sequences. However, as the sequences have been obtained from a purely metagenomic approach it is not possible to know which S, M and L segments belong together, for this viral isolation would be required.

One other interesting finding, especially considering the association of the tick and bats, was the presence of viral sequences belonging to the family *Rhabdoviridae*. These are non-segmented negative-sense RNA viruses known to infect a wide range of hosts, including, vertebrates, invertebrates and plants [28]. Of major public health concern are those belonging to the lyssavirus genus including, rabies virus and European bat lyssavirus 1 and 2 [29]. The rhabdoviral sequences from this study was highly divergent showing only around 30% to known rhabdoviruses and did not group together with lyssaviruses in the phylogenetic analysis indicating that they do not belong to that particular genus. Instead, phylogenetically, they grouped together with Tacheng tick virus 7, which is a virus that was discovered in ticks in a large arthropod viral metagenomic investigation [30].

Sequences matching to all segments of thogotoviruses were also identified. These are segmented viruses belonging to the *Orthomyxoviridae* and are known to be transmitted to vertebrates by ticks [31]. A number of viruses within this genus, such as, Dhori, Thogoto, and Bourbon viruses have been shown to infect and even cause disease in humans and animals. Phylogenetic analysis of the complete matrix protein grouped our sequence with the mentioned thogotoviruses rather than with other *Orthomyxoviridae* members. Depending on the segment investigated the a.a. identity varied between 47–97% to Dhori thogoto virus indicating that the thogotovirus in these ticks most likely belong to its own species within the *thogotovirus* genus.

An additional finding that could be of interest from a veterinary and public health perspective is the presence of sequences being classified as members of the *Reoviridae* and particularly within the rotavirus genus. Rotaviruses are globally spread viruses that cause gastroenteritis and in development countries they are a major cause of death among young children [32]. These are double-stranded segmented RNA viruses and the rotaviral sequences from this study matched to all the 11 expected genome segments [33]. The closest similarity of the rotaviral sequences from our study were to human and porcine rotaviruses, however, it was significant sequence differences and it is therefore not possible to conclude if this virus could infect humans and/or domestic animals such as pigs. Rotavirus is not considered a vector-borne virus transmitted by ticks, however, it is known that bats can harbour rotaviruses and it is therefore most likely that ticks have ingested rotavirus while feeding on the bat guano but are probably not being infected by/transmitting the rotavirus.

In addition, to the above discussed viruses a number of other highly divergent viruses were identified in the samples. For many of the viruses identified the similarity to other viruses was low, even on protein level, indicating that these might represent novel viruses within previously uncharacterised genera.

Taken together, the virome of the *A. delacruzi* ticks have both similarities and differences to those reported from other tick species and from other parts of the world. Vandergrift, K. and Kapoor A. (2019) investigated eight viral metagenomic articles and compared the virome of ticks collected from five different countries (United states, Norway, France, Australia and China) and concluded that flaviviruses were the most common positive-sense RNA virus [26]. Unlike those studies and the study from Brazil investigating the viral diversity of *Rhipicephalus microplus* [5] no flaviviruses were identified in our study. The most common negative-sense RNA viruses, from the five mentioned countries, belonged to the orders *Bunyavirales* and *Mononegavirales* and more specifically to the families *Chuviridae*, *Rhabdoviridae*, *Phenuviridae*, *Nairoviridae* and *Orthomyxoviridae*. These results are similar to ours as the main negative-sense RNA viruses in our study belong to the families *Nairoviridae*, *Orthomyxoviridae* and *Rhabdoviridae.* However, we could not identify any member belonging to the *Chuviridae* but it is known that viruses from this family are present in Brazil as it has been identified in *Rhipicephalus microplus* ticks collected in the southern part of the country [5]. The only double-stranded RNA viruses that have been identified in ticks all belong to the genus *Colitvirus* of the family *Reoviridae*, however, in our study members of the genus *Rotavirus* were identified. But as discussed previously this finding may reflect the special feeding properties of these ticks.

Further studies are needed to determine the host specificity and possible transmission routes of these different viruses between the ticks and bats. Considering the shared ecological niche of the *A. delacruzi* ticks and the bats it is interesting to speculate whether the bats could act as a reservoir for tick-borne viruses and potentially also being directly involved in spreading these viruses to other mammals. In addition, as the *A. delacruzi* ticks in the adult and larvae stage feed on bat guano rather than taking a blood meal it would mean that they could potentially ingest and be infected by viruses being shed through the faeces and is, thus, not dependent on a viremic bat. Attempting viral isolation as well as simultaneous viral investigations of ticks (adult and larvae), of bat guano and of blood samples from bats in the same cave would be a possible approach to further investigate the origin, host specificity and ecology of the identified viruses. 

## Figures and Tables

**Table 1:** Viral annotation. The main viral families identified through the blastx analysis. The table show those families with more than 500 reads assigned to them.

Viral Type	Viral Family	Number of Assigned Reads	% of Viral Reads
Single-stranded negative-sense RNA virus	*Nairoviridae*	753,197	88.2
	*Peribunyaviridae*	10,515	1.2
	*Rhabdoviridae*	11,075	1.3
	*Orthomyxoviridae*	17,553	2.1
Single-stranded positive-sense RNA virus	*Iflaviridae*	14,040	1.6
	*Dicistroviridae*	10,224	1.2
	*Polycipiviridae*	1271	0.1
Double-stranded RNA virus	*Reoviridae*	702	0.1
Unclassified viruses	Unclassified viruses	32,371	3.8

**Table 2:** Protein coverage and a.a. identity in relation to Dhori thogotovirus.

Segment	Protein	% Coverage	% a.a. Identity
1	PB2	68	97
2	PB1	92	82
3	PA	47	54
4	NP	73	61
5	GP	38	53
6	M	100	47

**Table 3:** Protein cover and a.a. identity in relation to the adult diarrheal rotavirus strain J19. “n.d.” marks that a specific protein was not detected in the analysis.

Segment	Protein	% Cover	% a.a. Identity
1	VP1	88.5	74–87
2	VP2	75.7	60–94
3	VP3	38.9	55–59
4	VP4	62.7	64
5	NSP1	67.3	52–57
6	VP6	67.9	84
7	NSP3	76.0	63
8	NSP2	92.6	79–81
9	VP7	54.7	63
10	NSP4	27.7	49–53
11	NSP5	87.5	61
11	NSP6	n.d.	n.d.
